# Association between the stress hyperglycemia ratio and all-cause mortality in patients with hemorrhagic stroke: a retrospective analysis based on MIMIC-IV database

**DOI:** 10.3389/fneur.2025.1526169

**Published:** 2025-05-29

**Authors:** Wei Zhu, Dingke Wen, Lijuan Duan, Chaofeng Fan, Yan Jiang

**Affiliations:** ^1^Department of Neurosurgery, West China Hospital, Sichuan University, Chengdu, China; ^2^West China School of Nursing, Sichuan University, Chengdu, China; ^3^Department of Nursing, West China Hospital, Sichuan University, Chengdu, China

**Keywords:** stress hyperglycemia ratio, stroke, Boruta algorithm, mortality, retrospective study

## Abstract

**Background:**

Research on the associations between the stress hyperglycemia ratio (SHR) and adverse outcomes in patients with hemorrhagic stroke is limited. Therefore, we aimed to investigate the relationship between the SHR and all-cause mortality in patients with hemorrhagic stroke.

**Methods:**

Clinical data of patients with hemorrhagic stroke were extracted from the Medical Information Mart for Intensive Care (MIMIC-IV) database. The patients were divided into four groups based on the SHR quartiles. Outcomes including 28-, 90-, and 365-day all-cause mortality were analyzed. Kaplan–Meier curves, Cox proportional hazard regression, and restricted cubic splines were used to investigate the relationships between the SHR and all-cause mortality. A machine learning prediction model integrating SHR was developed to assess its prognostic value for all-cause mortality.

**Results:**

The final analysis cohort consisted of 939 patients. Compared to the lowest SHR quartile, the highest quartile had significantly increased mortality risks at 28 days [hazard ratio (HR) = 4.53, 95% CI: 2.75–7.46; *p* < 0.001], 90 days (HR = 3.29, 2.19–4.95; *p* < 0.001), and 365 days (HR = 2.25, 1.60–3.17; *p* < 0.001). A significant upward trend in mortality risk was observed across ascending SHR quartiles (*p*-trend < 0.001 for all time points). Restricted cubic spline analysis demonstrated non-linear associations between SHR and all-cause mortality at 28 and 90 days (*p*-non-linear < 0.05), while the overall trend remained significantly positive. The machine learning models identified SHR as a key predictor, with area under the curves (AUC) of 0.771 (28-day), 0.778 (90-day), and 0.778 (365-day).

**Conclusion:**

This study revealed threshold-dependent associations between the SHR and short- and long-term all-cause mortality in patients with hemorrhagic stroke. The SHR was a reliable predictor for adverse outcomes in patients with hemorrhagic stroke.

## 1 Introduction

Stroke remains the second leading cause of death and a critical threat to public health globally (1). Hemorrhagic stroke, comprising intracerebral hemorrhage (ICH) and subarachnoid hemorrhage, is a particularly severe subtype of stroke with mortality rates as high as 45%−50% in the first 30 days (2, 3). In China, there were 1.07 million new hemorrhagic stroke cases and 1.16 million hemorrhagic stroke deaths in 2019, accounting for 27.1% of new strokes and 53% of stroke deaths (4). In recent decades, considerable efforts have been made to treat and care for hemorrhagic stroke. However, the availability of ideal treatment options to improve patient prognosis remains limited. Therefore, the identification of modifiable prognostic factors for individuals with hemorrhagic stroke is crucial for providing insights into potential therapeutic strategies.

Stress hyperglycemia, which refers to a transient increase in blood glucose levels in response to acute physiological stressors (5), is common among patients experiencing hemorrhagic stroke. After the occurrence of a stroke, activation of the sympathetic nervous system and the hypothalamic-pituitary-adrenal axis leads to the release of catecholamines and cortisol (5, 6). These increased hormone levels subsequently inhibit insulin secretion, promote glycogenolysis, and stimulate hepatic gluconeogenesis, resulting in a temporary elevation in blood glucose levels (7). Research has indicated that ~60% of patients with intracerebral hemorrhage experience hyperglycemia, and over 70% of patients with subarachnoid hemorrhage also exhibit elevated blood glucose levels as well (8, 9). Previous studies have extensively documented the associations between stress hyperglycemia and adverse outcomes, such as prolonged hospital stays, poor functional recovery, and increased mortality (10, 11). In these studies, stress hyperglycemia is indicated by admission blood glucose levels in hemorrhagic stroke patients.

However, in cases of hemorrhagic stroke, the sole reliance on absolute admission blood glucose levels proves insufficient to distinguish between a physiological stress response and inadequate background glycemic control. Because diabetes mellitus is a well-established risk factor for stroke (12). To address this issue, Roberts et al. (13) introduced the stress hyperglycemia ratio (SHR), a novel index integrating fasting blood glucose with glycosylated hemoglobin (HbA1c, reflecting glycemic status over 2–3 months), to better assess stress-induced hyperglycemia. The SHR has emerged as a critical tool for risk stratification, enabling clinicians to identify patients at higher risk of complications such as hematoma expansion, and tailor glycemic management strategies (e.g., intensive glucose monitoring or targeted insulin therapy) (14, 15). For example, in acute care settings, SHR thresholds >1.31 has been associated with a 3.3-fold increase in 30-day mortality risk, prompting earlier intervention in such cases (15). Several studies have investigated the correlations between the SHR and patient outcomes in hemorrhagic stroke patients. Notably, Chu et al. (14) have revealed that the SHR is a reliable predictor of hematoma expansion and unfavorable outcomes, such as secondary neurological decline and poor functional recovery at 3 months, among patients with spontaneous intracerebral hemorrhage. Liang et al. (15) further highlighted the SHR as a risk factor for 30-day and 1-year mortality in this patient population. Similarly, Li et al. (16) reported that the SHR is associated with both short- and long-term functional outcomes in patients with intracerebral hemorrhage. Despite of these advances in the knowledge of SHR, three critical gaps persist in the literature. First, previous studies have focused exclusively on intracerebral hemorrhage, while subarachnoid hemorrhage, a subtype with distinct pathophysiology and high prevalence of stress hyperglycemia, remains understudied. Second, conflicting results have been reported in previous studies investigating the interactions between diabetes mellitus, the SHR, and patient outcomes (15, 17), indicating the need for further clarification of the associations between the SHR and the prognosis of hemorrhagic stroke patients, considering their varied glucose metabolism statuses. Third, no prior research has systematically compared the predictive performance of SHR against established prognostic markers in hemorrhagic stroke populations.

Therefore, our aim was to investigate the associations between the SHR and all-cause mortality in patients with hemorrhagic stroke stratified by the presence of diabetes mellitus and other conditions, and to clarify the importance of the SHR in predicting prognosis. The findings of this study may contribute to a better understanding of the impact of stress hyperglycemia on the outcomes of patients with hemorrhagic stroke, and potentially inform the development of new strategies for risk stratification and management in stress hyperglycemia.

## 2 Materials and methods

### 2.1 Study design and population

The Medical Information Mart for Intensive Care (MIMIC-IV, version 2.2), a comprehensive and publicly accessible critical care database maintained by the Computational Physiology Laboratory at the Massachusetts Institute of Technology (MIT), served as the primary data source for this retrospective study. This database comprises ~73,000 detailed records of intensive care unit (ICU) stays at Beth Israel Deaconess Medical Center (18). Patients with hemorrhagic stroke were recruited for this study based on the International Classification of Diseases, Ninth Revision (ICD-9) and Tenth Revision (ICD-10). Specifically, for non-traumatic subarachnoid hemorrhage, the following ICD codes were selected: ICD-9 code 430, ICD-10 codes I60, I601-I609, I600, I6000-I6002, I6010-I6012, I6020-I6022, I6030-I6032, and I6050-I6052. For intracerebral hemorrhage, the study included the ICD-10 codes I61, I610-I619, I62, and I629, in addition to the ICD-9 codes 431, 4329, 7670, and a range of 77,210–77,214. Patients were excluded if they were: (1) under 18 years of age; (2) had ICU stays shorter than 3 h; or (3) lacked records of fasting blood glucose or glycated hemoglobin within 24 h following ICU admission. Furthermore, in cases where patients had multiple ICU admissions, only the initial record was considered.

### 2.2 Data extraction

**Figure 1:**
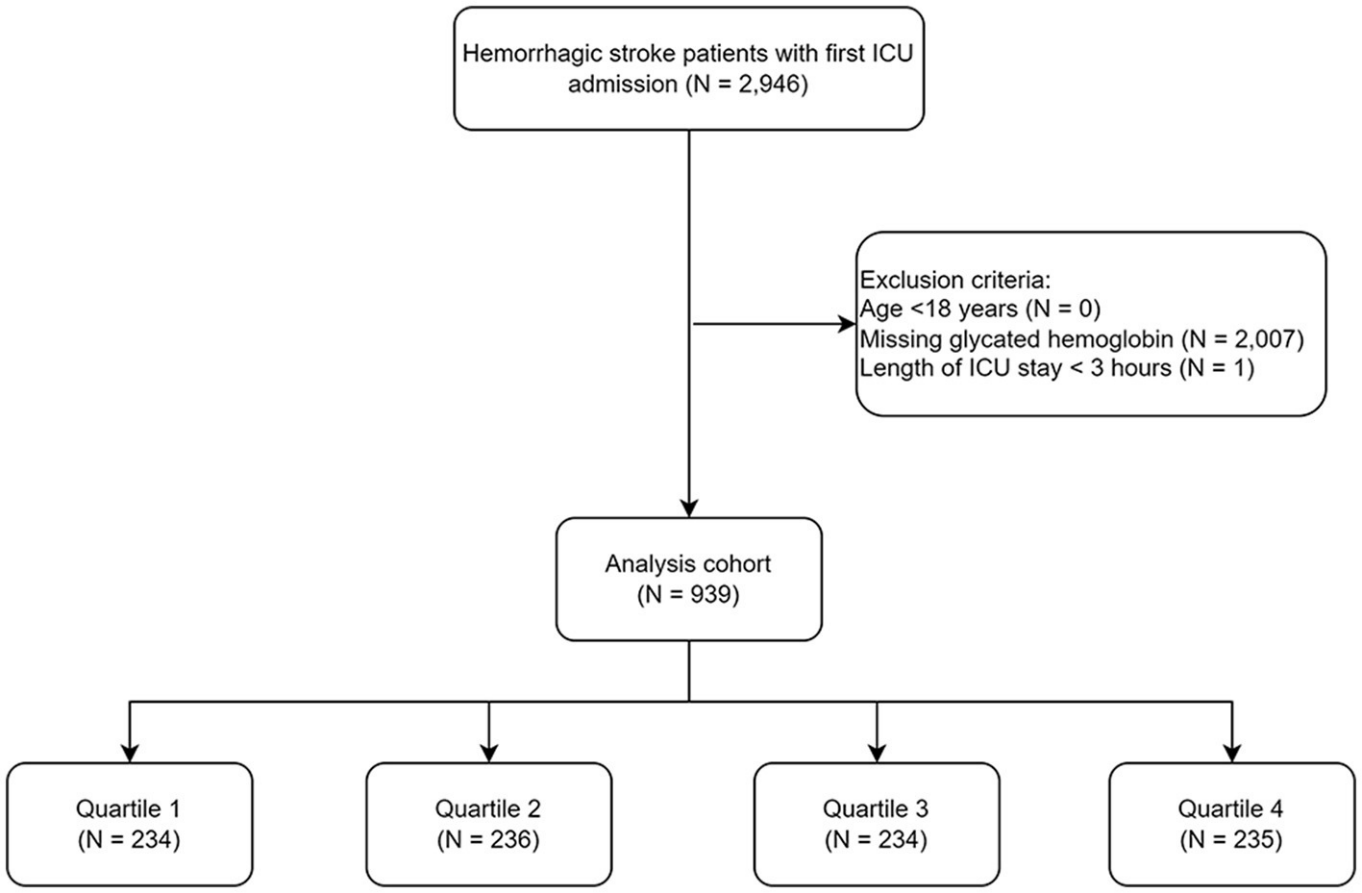
Flow chart of the patients enrolled throughout the trial. ICU, intensive care unit.

To access the data, one of the authors, Wei Zhu, completed a training course provided by the National Institutes of Health (NIH) to protect human study participants and obtained certification from the Collaborative Institutional Training Initiative (Record ID: 62,749,768). Data retrieval was performed via Navicat Premium software (version 17), which employed structured query language to extract information from five distinct categories: sociodemographic variables, clinical characteristics, comorbidities, laboratory parameters, and vital signs. Sociodemographic variables included age, sex, and weight. Clinical characteristics consisted of the Glasgow Coma Scale (GCS) score, Sequential Organ Failure Assessment (SOFA) score, and diagnosis. The comorbidity list included hypertension, diabetes, heart failure (HF), peripheral vascular disease (PVD), chronic pulmonary disease (COPD), malignant cancer, renal disease, and the Charlson Comorbidity Index (CCI). The laboratory parameters included red blood cell count (RBC), hemoglobin (Hb), white blood cell count (WBC), platelet count (PLT), serum sodium, potassium, calcium, chloride, anion gap, bicarbonate, creatinine, blood urea nitrogen (BUN), international normalized ratio (INR), prothrombin time (PT), activated partial thromboplastin time (APTT), fasting blood glucose (FBG), and HbA1c. Vital signs including heart rate (HR), systolic blood pressure (SBP), diastolic blood pressure (DBP), peripheral oxygen saturation (SpO_2_), temperature, and respiratory rate, were also recorded. The SHR was calculated via the following formula: SHR = FBG (mmol/L)/[1.59 × HbA1c (%)−2.59] (13, 19). The detailed data screening process was summarized in Figure 1. Briefly, from the MIMIC-IV database, we initially identified 2,946 adult hemorrhagic stroke patients with first ICU admission based on predefined ICD codes. After excluding patients those with missing HbA1c (*N* = 2,007), and length of ICU stays < 3 h (*N* = 1), 939 patients were included in the final analysis.

### 2.3 Outcomes

The primary outcome of this study was 28-day all-cause mortality of patients with hemorrhagic stroke. The secondary outcomes were 90- and 365-day all-cause mortality.

### 2.4 Data analysis

Variables with more than 20% missing data were excluded prior to the final analysis. For the remaining variables, missingness ranged from 0.2 to 18.7% (see Supplementary Table S1). The Little's missing completely at random test was performed, revealing a missing at random (MAR) pattern (Chi-square = 1,652.901, df = 1,030, *p* < 0.001). Given the MAR mechanism, the expectation maximization (EM) algorithm was chosen over alternative methods due to its statistical rigor in maximizing observed data likelihood, its ability to preserve multivariate covariance structures (unlike mean imputation which introduces bias), and its computational efficiency compared to resource-intensive approaches such as multiple imputation by chained (20). Missing data were subsequently imputed using the EM algorithm. To identify multicollinearity, the variance inflation factor was calculated for all variables. Patients were divided into four groups based on the SHR quartiles. Continuous variables were summarized using means and standard deviations (SDs) for normally distributed data, and median and interquartile ranges (IQRs) for non-normally distributed data. Categorical variables were presented as frequencies and percentages. To compare variables across SHR-stratified groups, the Kruskal–Wallis test and Chi-square test were performed.

The Kaplan–Meier survival curves were used to compare survival rates among the four groups stratified by quartiles of the SHR, with the log-rank test used to assess statistical significance. A multivariate Cox proportional hazard regression analysis was then conducted to estimate the hazard ratio (HR) and 95% confidence interval (95% CI) for the occurrence of the event. Variables that were significant in the univariate analysis and had clinical significance were included in the multivariate model. Model 1 served as the unadjusted model, whereas Model 2 was adjusted for age, sex, diagnosis, hypertension, diabetes, COPD, CCI, SOFA scores, WBC, anion gap, bicarbonate, BUN, calcium, chloride, potassium, APTT, and PLT. The restricted cubic spline (RCS) method was applied to investigate the non-linear relationship between the SHR and all-cause mortality through Cox proportional hazard models and to identify the optimal threshold. The optimal number and placement of knots in the RCS were determined by minimizing the Akaike information criterion. Two-segment Cox proportional risk models were subsequently applied on both sides of the inflection point to further evaluate the associations between the SHR and mortality risk. Additionally, stratification and interaction analyses were performed to dissect the impact of factors such as sex, age (dichotomized as below or above 65 years), the presence of diabetes and hypertension, and diagnosis on the relationship between the SHR and mortality risk. For each subgroup analysis, Cox proportional hazard regression analyses were employed, adjusting for the same confounders as in Model 2. Likelihood ratio tests were utilized to identify significant interactions.

Boruta's algorithm can determine features that are most important for predicting the target variable by simulating randomness (21, 22). It was also utilized to select features for establishing a mortality prediction model and to determine the importance of the SHR as a predictor. The dataset was randomly divided into a training set and a validation set at a ratio of 7:3 for model establishment and evaluation, respectively. The variables identified by Boruta's algorithm were integrated into the Cox proportional hazard survival learner (*coxph*) algorithm to predict all-cause mortality at 28, 90, and 365 days. The ROC curve and its corresponding area under the curve (AUC) were utilized to assess model performance, and decision curve analysis (DCA) was employed to evaluate clinical effectiveness. The calibration curves were used to assess the accuracy of the model in predicting absolute risk. All the statistical analyses were conducted via R software (version 4.2.2) and SPSS 24.0 (IBM SPSS Statistics, Armonk, NY, USA). A two-tailed *p* value of < 0.05 was considered statistically significant.

## 3 Results

### 3.1 Baseline characteristics

Table 1 displays the baseline characteristics of the included patients stratified by SHR quartiles [quartile 1 (SHR < 0.90), quartile 2 (0.90 ≤ SHR < 1.05), quartile 3 (1.05 ≤ SHR < 1.26), and quartile 3 (SHR > 1.26)]. The median age of the patients in this study was 71.0 years (IQR: 60.0–82.0), and 53.6% of them were male. Significant differences were identified in age, SOFA scores, diagnosis, presence of diabetes, CCI scores, WBC, serum sodium, anion gap, bicarbonate, chloride, FBG, HbA1c, HR, respiratory rate, body temperature, and SpO_2_ across patients stratified by the SHR quartiles.

**Table 1:** Baseline characteristics and outcomes of patients stratified by SHR.

**Characteristics**	**Overall (*N* = 939)**	**Q1 (*N* = 234)**	**Q2 (*N* = 236)**	**Q3 (*N* = 234)**	**Q4 (*N* = 235)**	***p*-value**
SHR	1.05 (0.90, 1.25)	0.81 (0.74, 0.86)	0.97 (0.93, 1.01)	1.14 (1.09, 1.19)	1.43 (1.35, 1.68)	< 0.001^†^
**Demographic variables**						
Age (year, IQR)	71.0 (60.0, 82.0)	73.0 (63.0, 83.0)	73.0 (61.8, 83.0)	70.0 (58.0, 80.0)	70.0 (58.0, 79.0)	0.007^†^
Male (*n*, %)	503 (53.6)	130 (55.6)	135 (57.2)	125 (53.4)	113 (48.1)	0.215^‡^
Weight (kg, IQR)	76.8 (63.8, 91.7)	76.9 (63.1, 89.2)	74.6 (63.4, 90.0)	80.0 (68.3, 94.8)	75.0 (61.0, 92.7)	0.112^†^
**Clinical characteristics**						
GCS	14 (12, 15)	14 (12, 15)	14 (12, 15)	14 (12, 15)	14 (11, 15)	0.939^†^
SOFA	3 (1, 4)	2 (1, 4)	2 (1, 4)	3 (1, 4)	3 (2, 5)	< 0.001^†^
ICH (*n*, %)	797 (84.9)	213 (91.0)	211 (89.4)	186 (79.5)	187 (79.6)	< 0.001^‡^
**Comorbidities**						
Hypertension (*n*, %)	681 (72.5)	162 (69.3)	169 (71.6)	174 (74.4)	176 (74.9)	0.488^‡^
Diabetes (*n*, %)	280 (29.8)	79 (33.8)	58 (24.6)	51 (21.8)	92 (39.2)	< 0.001^‡^
HF (*n*, %)	141 (15.0)	43 (18.4)	36 (15.3)	28 (12.0)	34 (14.5)	0.279^‡^
PVD (*n*, %)	63 (6.7)	20 (8.6)	19 (8.1)	17 (7.3)	7 (3.0)	0.063^‡^
COPD (*n*, %)	115 (12.3)	28 (12.0)	25 (10.6)	26 (11.1)	36 (15.3)	0.397^‡^
Malignant cancer (*n*, %)	47 (5.0)	12 (5.1)	10 (4.2)	13 (5.6)	12 (5.1)	0.929^‡^
Renal disease (*n*, %)	123 (13.1)	42 (18.0)	31 (13.1)	23 (9.8)	27 (11.5)	0.056^‡^
CCI	6 (4, 8)	6 (4, 8)	6 (4, 8)	5 (4, 7)	6 (4, 8)	0.010^†^
**Laboratory parameters**						
RBC (10^9^/L)	4.2 (3.7, 4.6)	4.2 (3.8, 4.6)	4.3 (3.8, 4.6)	4.2 (3.8, 4.5)	4.1 (3.6, 4.5)	0.088^†^
Hb (g/L)	12.6 (11.3, 13.8)	12.5 (11.1, 13.8)	12.7 (11.5, 13.9)	12.6 (11.7, 13.8)	12.4 (10.9, 13.8)	0.087^†^
WBC (10^9^/L)	9.8 (7.8, 12.4)	8.7 (7.1, 10.7)	9.3 (7.2, 12.1)	10.4 (8.6, 12.9)	11.4 (8.8, 14.3)	< 0.001^†^
PLT (10^9^/L)	211 (171, 259)	214 (177, 253)	209 (170, 260)	205 (170, 264)	208 (169, 259)	0.912^†^
Sodium (mmol/L)	140 (137, 142)	140 (138, 143)	139 (138, 142)	140 (137, 142)	139 (137, 141)	< 0.001^†^
Potassium (mmol/L)	4.0 (3.7, 4.3)	4.0 (3.6, 4.3)	4.0 (3.7, 4.3)	3.9 (3.7, 4.3)	4.0 (3.7, 4.4)	0.242^†^
Calcium (mmol/L)	8.9 (8.5, 9.2)	8.9 (8.5, 9.2)	8.9 (8.6, 9.2)	8.9 (8.5, 9.2)	8.9 (8.4, 9.2)	0.773^†^
Creatinine (mg/dl)	0.9 (0.7, 1.1)	0.9 (0.7, 1.1)	0.9 (0.7, 1.0)	0.9 (0.7, 1.1)	0.9 (0.8, 1.2)	0.108^†^
Bun (mg/dl)	17.0 (13.0, 21.9)	16.0 (12.0, 21.8)	17.0 (13.0, 21.0)	16.0 (12.3, 20.0)	18.0 (13.0, 23.0)	0.069^†^
Anion gap (mmol/L)	15 (13, 17)	14 (12, 16)	14 (13, 16)	15 (13, 16)	16 (14, 18)	< 0.001^†^
Bicarbonate (mmol/L)	24 (22, 26)	24 (22, 26)	24 (22, 26)	24 (22, 26)	22 (21, 24)	< 0.001^†^
Chloride (mmol/L)	103 (101, 106)	104 (102, 107)	103 (10, 105)	103 (101, 106)	103 (100, 106)	< 0.001^†^
INR	1.1 (1.1, 1.2)	1.1 (1.1, 1.3)	1.1 (1.1, 1.2)	1.1 (1.1, 1.2)	1.1 (1.0, 1.3)	0.682^†^
PT (s)	12.4 (11.5, 13.8)	12.5 (11.6, 13.8)	12.3 (11.6, 13.7)	12.5 (11.6, 13.6)	12.3 (11.3, 14.1)	0.384^†^
APTT (s)	28.1 (25.8, 30.8)	28.3 (26.0, 30.7)	28.4 (26.2, 30.9)	27.6 (25.3, 30.2)	28.0 (25.4, 31.0)	0.257^†^
FBG (mmol/L)	6.9 (5.8, 8.6)	5.4 (5.1, 6.1)	6.3 (5.6, 7.0)	7.4 (6.7, 8.1)	9.6 (8.3, 12.5)	< 0.001^†^
HbA1c (%)	5.7 (5.4, 6.3)	5.9 (5.6, 6.5)	5.7 (5.4, 6.1)	5.6 (5.3, 6.1)	5.6 (5.2, 6.6)	< 0.001^†^
**Vital signs**						
HR (beats/min)	78 (70, 87)	74 (68, 85)	77 (70, 87)	78 (69, 87)	83 (74, 93)	< 0.001^†^
SBP (mmHg)	133 (124, 142)	133 (124, 142)	135 (125, 142)	134 (124, 142)	132 (125, 138)	0.513^†^
DBP (mmHg)	69 (62, 77)	70 (63, 77)	68 (62, 78)	69 (63, 76)	67 (59, 75)	0.036^†^
Respiratory rate	18 (17, 20)	18 (16, 20)	18 (17, 20)	18 (16, 20)	19 (17, 21)	< 0.001^†^
Temperature (°C)	36.9 (36.7, 37.2)	36.9 (36.7, 37.1)	36.9 (36.7, 37.2)	37.0 (36.8, 37.2)	37.0 (36.8, 37.3)	0.010^†^
SpO_2_ (%)	97 (96, 98)	97 (96, 98)	97 (96, 98)	97 (96, 98)	98 (96, 99)	0.001^†^
**Outcomes**						
In-hospital ACM	149 (15.9)	19 (8.1)	32 (13.6)	27 (11.5)	71 (30.2)	< 0.001^‡^
28 days ACM	187 (19.9)	24 (10.3)	39 (16.5)	41 (17.5)	83 (35.3)	< 0.001^‡^
90 days ACM	240 (25.6)	38 (16.2)	52 (22.0)	55 (23.5)	95 (40.4)	< 0.001^‡^
1 year ACM	295 (31.4)	61 (26.1)	68 (28.8)	64 (27.4)	102 (43.4)	< 0.001^‡^

Q1 (SHR < 0.90), Q2 (0.90 ≤ SHR < 1.05), Q3 (1.05 ≤ SHR < 1.26), Q4 (SHR > 1.26).

SHR, stress hyperglycemia ratio; GCS, Glasgow Coma Scale; SOFA, Sequential Organ Failure Assessment; ICH, intracranial hemorrhage; CCI, Charlson comorbidity index; HF, Heart failure; PVD, Peripheral vascular disease; COPD, chronic pulmonary disease; RBC, red blood cell count; Hb, hemoglobin; WBC, white blood cell count; PLT, platelet; BUN, blood urea nitrogen; INR, international normalized ratio; PT, prothrombin time; APTT, activated partial thromboplastin time; FBG, fasting blood glucose; HbA1c, glycated hemoglobin; HR, heart rate; SBP, systolic blood pressure; DBP, diastolic blood pressure; SpO_2_, saturation of peripheral oxygen; ACM, all-cause mortality.

^†^Kruskal–Wallis rank sum test.

^‡^Pearson's Chi-squared test.

### 3.2 Clinical outcomes

**Figure 2:**
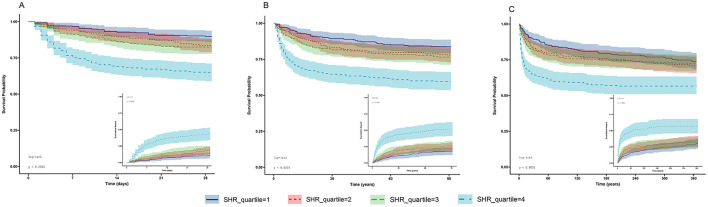
Kaplan–Meier survival analysis curves for **(A)** 28-day, **(B)** 90-day, and **(C)** 365-day all-cause mortality; stress hyperglycemia ratio (SHR) quartile 1 (SHR < 0.90), quartile 2 (0.90 ≤ SHR < 1.05), quartile 3 (1.05 ≤ SHR < 1.26), and quartile 4 (SHR > 1.26).

The overall mortality rates in this cohort progressively increased from 15.9% in the hospital to 19.9% at 28 days, 25.6% at 90 days, and culminated at 31.4% at 365 days. Furthermore, significant changes in mortality rates were observed among patients stratified by quartiles of the SHR. The Kaplan–Meier survival analysis underscored significant disparities in all-cause mortality at 28, 90, and 365 days among patients stratified by the SHR quartiles (Figure 2). Notably, patients in the SHR Quartile 4 presented the lowest survival probability at all the assessed time points (28, 90, and 365 days), with statistical significance confirmed by log-rank tests (*p* < 0.001). Table 2 shows the outcomes of the Cox proportional hazard regression models. High SHR, treated as a continuous variable, was significantly associated with an elevated risk of all-cause mortality at 28 days (HR = 3.06, 95% CI: 2.20–4.26, *p* < 0.001), 90 days (HR =2.54, 95% CI: 1.87–3.43, *p* < 0.001), and 365 days (HR = 2.09, 95% CI: 1.56–2.78, *p* < 0.001). Additionally, when compared to patients with an SHR of < 0.90 (Quartile 1), those with a higher quartile of SHR had an increased risk of all-cause mortality at both 28 and 90 days (*p* < 0.05). Similarly, patients with an SHR of >1.26 (Quartile 4) had an increased risk of 365-day (HR = 2.25, 95% CI: 1.60–3.17, *p* < 0.001) all-cause mortality compared with Quartile 1.

**Table 2:** Cox proportional hazard ratios for ACM at 28, 90, and 365 days.

**SHR**	**Unadjusted model**			**Adjusted model**		
	**HR**	**95% CI**	***p*** **-value**	**HR**	**95% CI**	***p*** **-value**
**28 days ACM**						
Continues variable per unit	3.18	2.47–4.10	< 0.001	3.06	2.20–4.26	< 0.001
**Quartile**						
Q1	Reference	–	–	Reference	–	–
Q2	1.65	0.99–2.74	0.053	2.02	1.19–3.44	0.01
Q3	1.79	1.08–2.97	0.023	2.18	1.28–3.71	0.004
Q4	4.19	2.66–6.59	< 0.001	4.53	2.75–7.46	< 0.001
*p* for trend			< 0.001			< 0.001
**90 days ACM**						
Continues variable per unit	2.82	2.22–3.59	< 0.001	2.54	1.87–3.43	< 0.001
**Quartile**						
Q1	Reference	–	–	Reference	–	–
Q2	1.41	0.92–2.14	0.111	1.68	1.09–2.59	0.019
Q3	1.53	1.01–2.32	0.043	1.85	1.20–2.85	0.005
Q4	3.12	2.14–4.54	< 0.001	3.29	2.19–4.95	< 0.001
*p* for trend			< 0.001			< 0.001
**365 days ACM**						
Continues variable per unit	2.31	1.81–2.95	< 0.001	2.09	1.56–2.78	< 0.001
**Quartile**						
Q1	Reference	–	–	Reference	–	–
Q2	1.15	0.81–1.62	0.431	1.35	0.95–1.93	0.097
Q3	1.11	0.78–1.58	0.557	1.34	0.93–1.94	0.113
Q4	2.12	1.54–2.91	< 0.001	2.25	1.60–3.17	< 0.001
*p* for trend			< 0.001			< 0.001

Q1 (SHR < 0.90), Q2 (0.90 ≤ SHR < 1.05), Q3 (1.05 ≤ SHR < 1.26), Q4 (SHR > 1.26).

SHR, stress hyperglycemia ratio; ACM, all-cause mortality; Adjusted model: adjusted for age, gender, diagnosis, hypertension, diabetes, COPD, CCI, SOFA scores, WBC, anion gap, bicarbonate, BUN, calcium, chloride, potassium, APTT, and PLT.

**Figure 3:**
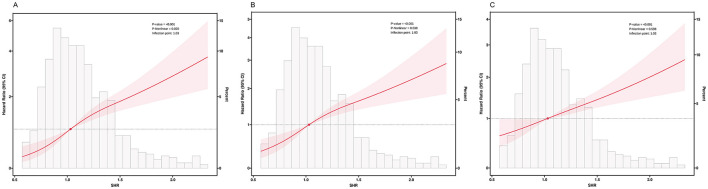
Restricted cubic spline curve for **(A)** 28-day, **(B)** 90-day, and **(C)** 365-day all-cause mortality; Curves represent estimated adjusted hazard ratios; shaded ribbons represent 95% confidence intervals; and red dot represents inflection point of the RCS curve; **(A)** The inflection point was identified at SHR = 1.03, *p*-non-linear = 0.020; **(B)** The inflection point was identified at SHR = 1.03, *p*-non-linear = 0.038; **(C)** The inflection point was identified at SHR = 1.03, *p*-non-linear = 0.598. HR, hazard ratio; CI, confidence interval; SHR, stress hyperglycemia ratio.

The RCS analysis results indicated significant non-linear associations between the SHR and mortality risk at 28 and 90 days, as illustrated in Figure 3. The inflection points in the non-linear relationship between the SHR and mortality risk at 28, 90, and 365 days were identified as 1.03. To further investigate these associations, two-segment Cox proportional hazard models were employed (see Supplementary Table S2). Specifically, when the SHR exceeded 1.03, a 1-unit increase in the SHR was associated with a 2.82-, 2.61-, and 2.39-fold elevated risks of 28-, 90-, and 365-day all-cause mortality (*p* < 0.001), respectively.

### 3.3 Subgroup analysis

**Figure 4:**
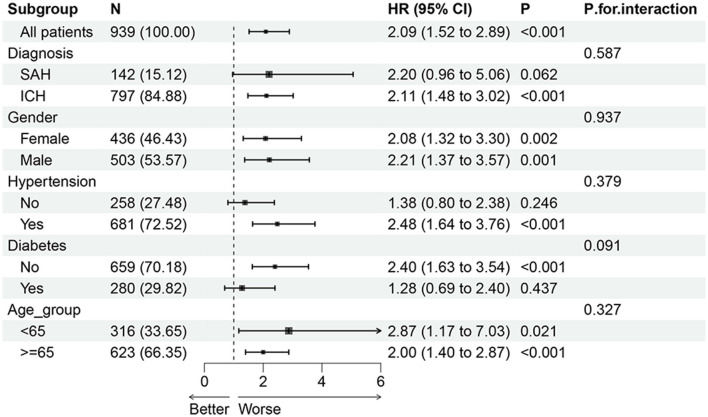
Subgroup forest plot for 28-day all-cause mortality; adjusted for age, sex, diagnosis, hypertension, diabetes, COPD, hypertension, diabetes, CCI, SOFA scores, WBC, anion gap, bicarbonate, BUN, calcium, chloride, potassium, APTT, and PLT. HR, hazard ratio; CI, confidence interval.

The associations between the SHR and mortality risk were further assessed in different patient subgroups, including age (below or over 65 years), sex, diagnosis, and the presence of hypertension and diabetes. The HRs were significant at 28 days (Figure 4), 90 days (Supplementary Figure S1), and 365 days (Supplementary Figure S2), regardless of age subgroup. The HRs were significant at 28 and 90 days regardless of the sex subgroup, while there was no statistically significant difference in male patients at 365 days. Additionally, the HRs were significant in patients with hypertension at all three time points. No statistically significant difference was observed in patients diagnosed with SAH at 28, 90, or 365 days. Notably, in patients without diabetes, the HRs were significant at 28 days (HR = 2.40, 95%CI: 1.63–3.54, *p* < 0.001), 90 days (HR = 1.90, 95%CI: 1.37–2.65, *p* < 0.001), and 365 days (HR = 1.60, 95%CI: 1.19–2.14, *p* = 0.002). In contrast, the HRs in the diabetic cohort demonstrated non-significant associations across all follow-up intervals.

### 3.4 Establishment and validation of the prediction model

**Figure 5:**
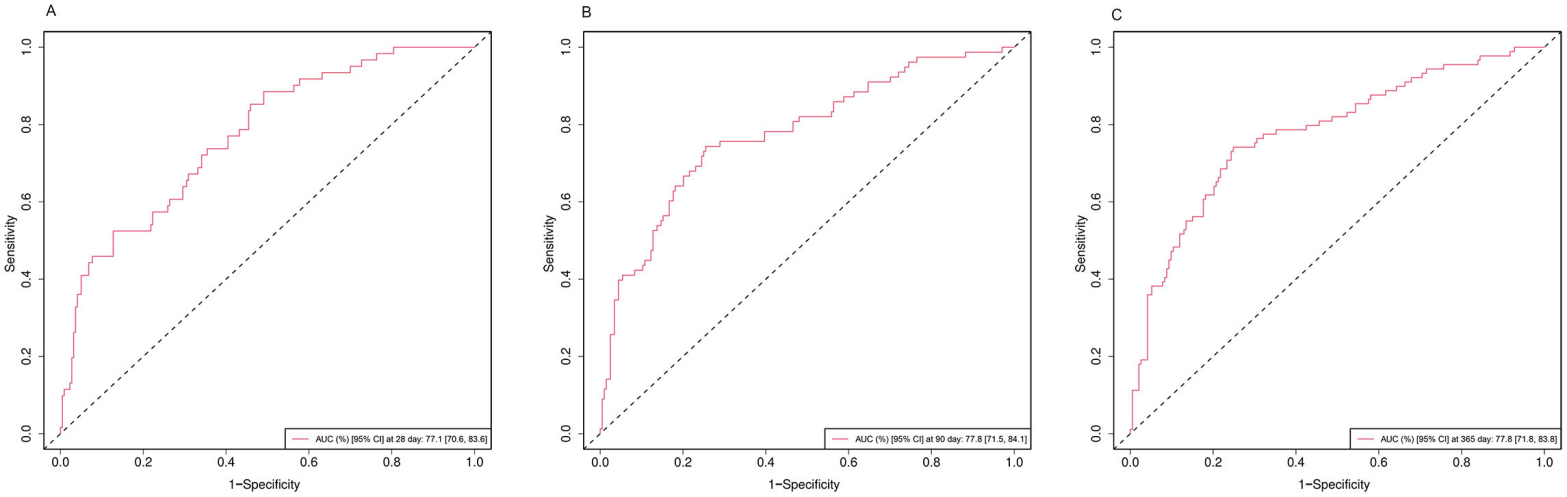
ROC curves of the prediction model for **(A)** 28-day, **(B)** 90-day, and **(C)** 365-day all-cause mortality. AUC, area under the curve; CI, confidence interval.

Supplementary Figure S3 displays the results of Boruta's algorithm. Variables within the green area were identified as important features and were used in the establishment of the prediction model. Figure 5 shows the ROC curves of the mortality prediction model. The AUC values were 0.771, 0.778, and 0.778 for the 28-, 90-, and 365- intervals, respectively. The calibration curves of each prediction model aligned well with the reference line, suggesting good predictive performance (Supplementary Figure S4). The DCA curves demonstrated net benefits at 28, 90, and 365 days, suggesting robust clinical validity for each model (Supplementary Figure S5).

## 4 Discussion

This study revealed that the SHR was associated with both short- and long-term all-cause mortality in patients with hemorrhagic stroke. Notably, a significant non-linear association was observed between the SHR and all-cause mortality, featuring an inflection point at 1.03. Furthermore, the SHR emerged as an important risk factor for all-cause mortality. The predictive model, which incorporated the SHR along with other established risk factors, showed good performance.

This study highlighted the significant associations between elevated SHR and increased all-cause mortality in patients with hemorrhagic stroke at various time intervals (28, 90, and 365 days). This finding aligns with those of previous studies that reported associations between the SHR and adverse outcomes in stroke patients (10, 23, 24). The potential mechanisms underlying how SHR contributes to these negative effects are complex and multifaceted. First, SHR is thought to be associated with increased oxidative stress and inflammatory responses. Neutrophil-derived reactive oxygen species (ROS) can activate the TLR4/MyD88/NF-κB pathway, promoting NLRP3 inflammasome assembly and IL-1β maturation (25). This cascade leads to endothelial dysfunction and neuronal apoptosis (26–28). Second, hyperglycemia can activate specific enzymes, such as protein kinase C and NADPH oxidase, further amplifying reactive oxygen species production and inhibiting nitric oxide synthase, leading to insufficient reperfusion and exacerbating neuronal injury (29). Moreover, SHR can compromise the structural integrity of blood vessels near the initial bleeding site, enhancing the expression of factors such as nuclear factor kappa B and matrix metalloproteinase-9 (30). These changes may contribute to hematoma expansion (14), a well-established predictive factor for poor prognosis in hemorrhagic stroke patients. Additionally, SHR may downregulate Aquaporin-4, a protein that helps prevent brain edema and protects blood-brain barrier (BBB) integrity (31, 32). In ischemic stroke rats, neutrophil-derived IL-1α/TNF induce Aquaporin-4 polarization to perivascular astrocyte end-feet, disrupting water homeostasis (33). This aggravates edema and blood-brain barrier (BBB) disruption. Finally, hyperglycemia can directly increase blood-brain barrier permeability, leading to edema and death of neuro-cells (34). Given these mechanisms, strategies to control blood glucose levels, reduce oxidative stress and inflammation, and protect BBB integrity could mitigate the negative effects of stress hyperglycemia and enhance patient outcomes.

In this study, we identified a significant non-linear association between the SHR and all-cause mortality at 28 and 90 days, with an inflection point at 1.03. This result is consistent with the outcomes reported by Liang et al. (15), who detected a similar inflection point of 1.04 for SHR in relation to 30-day mortality among patients with spontaneous intracerebral hemorrhage. The significance of glucose monitoring and management as pivotal components of comprehensive care for improving prognosis of hemorrhagic stroke patients is widely acknowledged (35). Nevertheless, the precise optimal range for blood glucose targets remains elusive. Ma et al. (36) conducted a multinational, multicenter, randomized controlled trial, and revealed that a bundled approach incorporating blood glucose management positively impacted 6-month outcomes for patients with intracerebral hemorrhage. Notably, their study set individualized blood glucose targets: 6.1–7.8 mmol/L for non-diabetic patients and 7.8–10.0 mmol/L for those with diabetes. Similarly, Middleton et al. (37) conducted a cluster randomized controlled trial, and demonstrated that a multifaceted intervention targeting temperature, glucose levels, and swallowing dysfunction led to improved outcomes in acute stroke patients. However, they adopted a target of 8.0 mmol/L for both diabetic and non-diabetic individuals. The aforementioned studies have determined target blood glucose levels based solely on absolute glucose values, which are inherently influenced by diabetic status and insulin sensitivity, potentially leading to bias in glycemic control. Consequently, the inflection points of SHRs, which take both the absolute blood glucose value and background glycemic level into account, may offer a new perspective on blood glucose management. Our subgroup analysis further revealed that diabetic patients had greater short- and long-term mortality risks than non-diabetic patients. This disparity could stem from diabetic patients' increased tolerance to hyperglycemia, as well as their routine insulin therapy, which may provide neuroprotective benefits through its anti-inflammatory properties, mitigating brain damage (38). Overlooking hyperglycemia in non-diabetic patients may also contribute to this discrepancy (15).

This study is the first to evaluate the importance of the SHR in predicting short- and long-term mortality risk in patients with hemorrhagic stroke. The results of the Boruta algorithm indicated that the SHR played a significant role in predicting all-cause mortality in patients with hemorrhagic stroke, along with other well-established risk factors, such as SOFA and GCS score. Moreover, this result suggests that the SHR is not a definitive decision factor, despite its importance in predicting mortality risk. By incorporating other acceptable variables selected by the Boruta algorithm, the models predicting all-cause mortality in patients with hemorrhagic stroke exhibited good performance. These findings align with previous studies in ischemic stroke. For example, Huang et al. (39) conducted a systematic review and meta-analysis that included 13 studies involving 184,179 acute ischemic stroke patients, and revealed that the SHR is a risk factor for functional outcomes and mortality. Similarly, Liang et al. (15) found SHR superior to admission blood glucose in predicting mortality in spontaneous intracerebral hemorrhage (ICH). Our study extends these observations by demonstrating SHR's predictive value in hemorrhagic stroke including both intracerebral hemorrhage and subarachnoid hemorrhage, while uncovering non-linear relationships between SHR levels and mortality risk. Importantly, SHR's accessibility and cost-effectiveness make it a practical tool for risk stratification in clinical settings.

However, this study is subject to several limitations. First, the limited nature of its retrospective observational design makes it impossible to establish causality. Although rigorous statistical adjustments were applied, residual confounding from unmeasured variables remains possible. Second, while EM algorithm was used to impute missing data in key variables, the accuracy of imputed values could not be clinically validated due to the lack of prospective data collection protocols, particularly for time-sensitive parameters such as blood glucose. Third, the use of blood glucose records from the initial ICU admission day as fasting glucose, in the absence of data to differentiate between random and fasting glucose levels, may introduce bias into the study findings. Finally, this study only used data from a single center, and the external validity of the findings is limited.

## 5 Conclusion

In conclusion, the SHR is an important and reliable indicator for mortality risk prediction in patients with hemorrhagic stroke. The optimal threshold of the SHR identified in this study may facilitate blood glucose monitoring and management and further improve patient prognosis. Future research should focus on conducting prospective, multicenter studies with larger cohorts to evaluate the clinical validity of this SHR threshold across diverse populations and healthcare settings. Such efforts will be crucial for establishing causal relationships and developing standardized clinical protocols.

## Data Availability

The raw data supporting the conclusions of this article will be made available by the authors, without undue reservation.
