# Supplementary material for: Association between the stress hyperglycemia ratio and all-cause mortality in patients with hemorrhagic stroke: a retrospective analysis based on MIMIC-IV database
Source: Front Neurol. 2025 May 29;16:1526169. doi: 10.3389/fneur.2025.1526169 (PMC12158693; doi:10.3389/fneur.2025.1526169)
Supplement: Supplementary file 1 [file Table_1.docx]

Supplementary Material

Table S1. Missing records of included variables

| Characteristics | Missing records (%) |
| --- | --- |
| SHR  **Demographic variables**  Age  Gender  Weight  **Clinical characteristics**  GCS  SOFA  Diagnosis  **Comorbidities**  Hypertension  Diabetes  HF  PVD  COPD  Malignant cancer  Renal disease  CCI  **Laboratory Parameters**  RBC  Hb  WBC  PLT  Sodium  Potassium  Calcium  Creatinine  Bun  Anion gap  Bicarbonate  Chloride  INR  PT  APTT  FBG  HbA1c  **Vital signs**  HR  SBP  DBP  Respiratory rate  Temperature  SpO_2_ | -  -  -  15(1.6)  2(0.2)  -  -  -  -  -  -  -  -  -  -  23(2.4)  23(2.4)  2(0.2)  17(1.8)  26(2.8)  40(4.3)  176(18.7)  38(4.0)  28(3.0)  41(4.4)  40(4.3)  37(3.9)  65(6.9)  77(8.2)  41(4.4)  -  -  -  -  -  -  - |

Table S2. Threshold effect analysis of SHR on 28-day, 90-day, and 365-day all-cause mortality

| Characteristic | HR | 95% CI | *P* value |
| --- | --- | --- | --- |
| **28-day ACM**  SHR (<1.03)  SHR (≥1.03)  **90-day ACM**  SHR (<1.03)  SHR (≥1.03)  **365-day ACM**  SHR (<1.03)  SHR (≥1.03) | 4.05  2.82  2.56  2.61  0.86  2.39 | 0.40-41.1  2.05-3.87  0.40-16.6  1.93-3.52  0.20-3.64  1.78-3.23 | 0.240  <0.001  0.320  <0.001  0.840  <0.001 |

HRs were adjusted for age, gender, diagnosis, hypertension, diabetes, COPD, hypertension, diabetes, CCI, SOFA scores, WBC, anion gap, bicarbonate, BUN, calcium, chloride, potassium, APTT, and PLT.


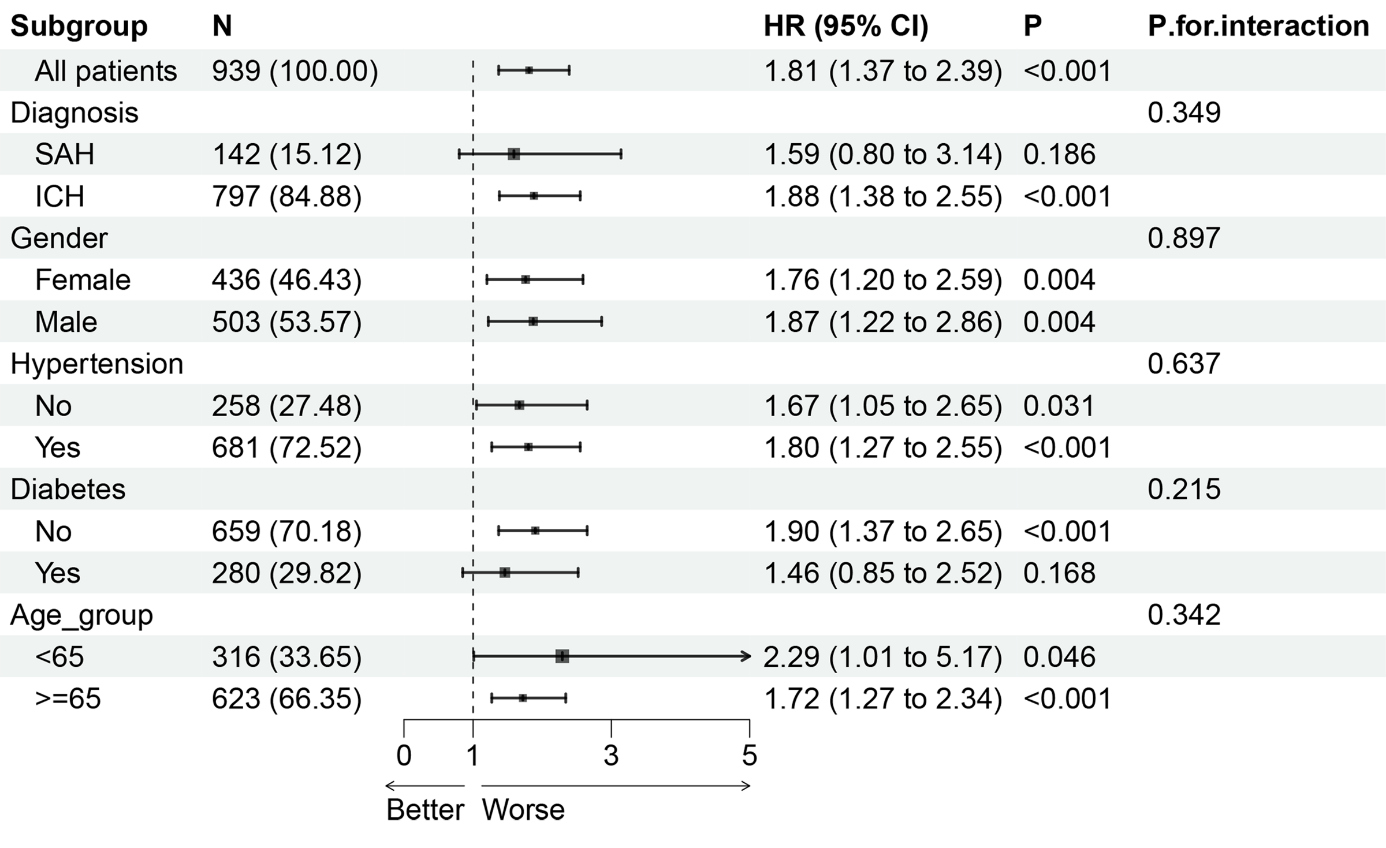


Figure S1 Forest plots of subgroup analyses of SHR and 90-day all-cause mortality. Adjusted for age, gender, diagnosis, hypertension, diabetes, COPD, hypertension, diabetes, CCI, SOFA scores, WBC, anion gap, bicarbonate, BUN, calcium, chloride, potassium, APTT, and PLT.


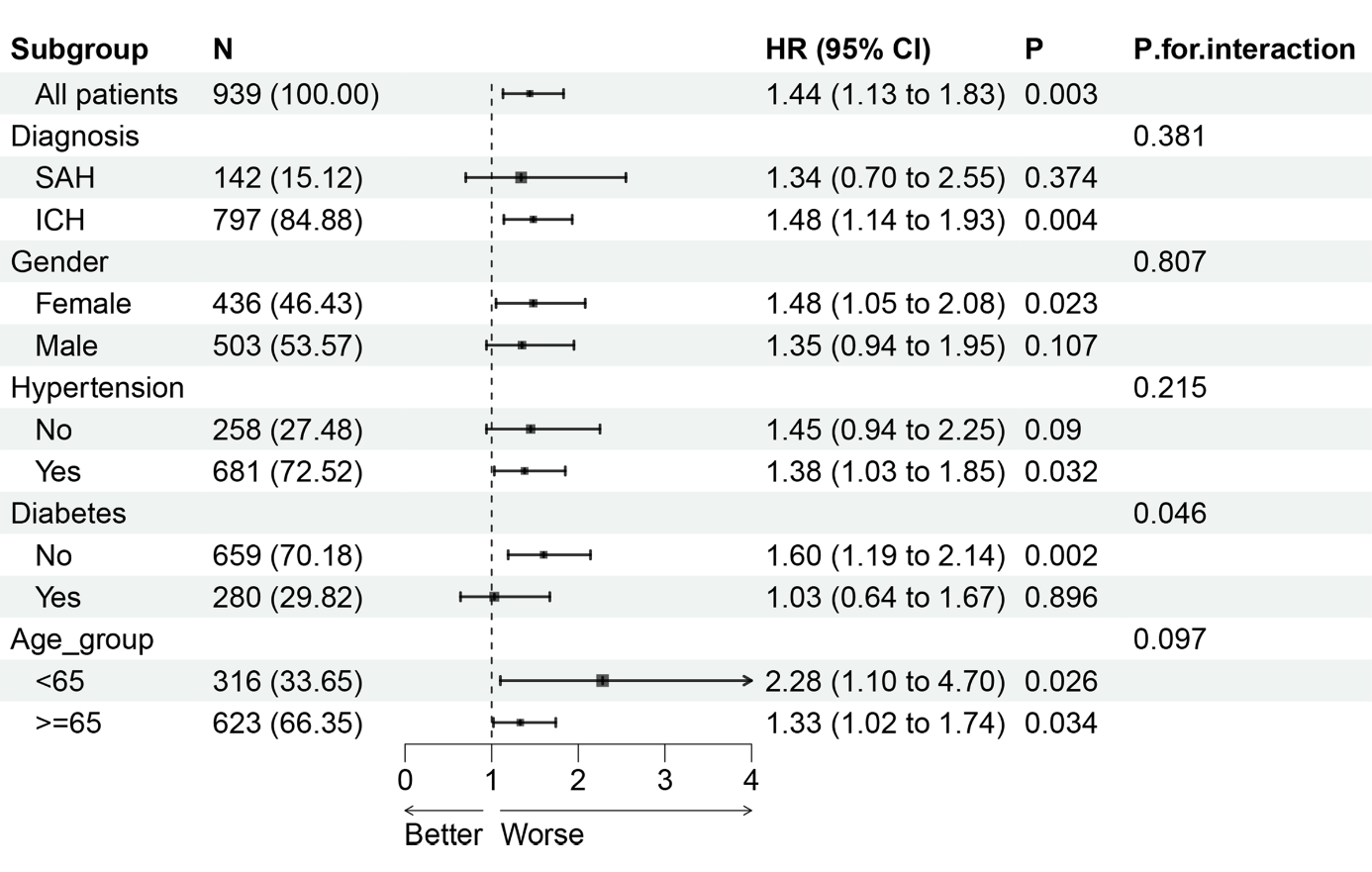


Figure S2 Forest plots of subgroup analyses of SHR and 365-day all-cause mortality. Adjusted for age, gender, diagnosis, hypertension, diabetes, COPD, hypertension, diabetes, CCI, SOFA scores, WBC, anion gap, bicarbonate, BUN, calcium, chloride, potassium, APTT, and PLT


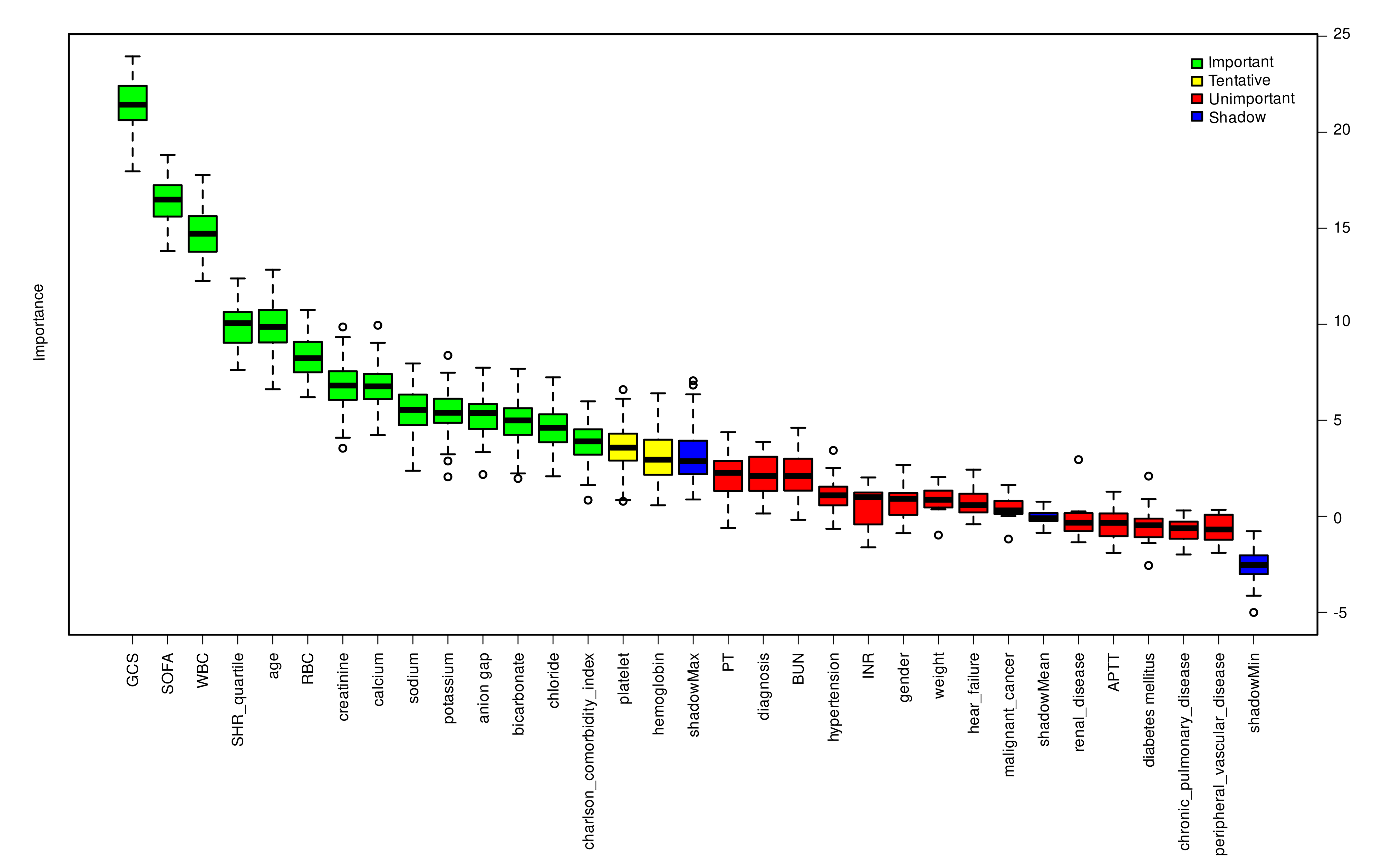


Figure S3. Feature selection based on the Boruta algorithm. The horizontal axis is the name of each variable, and the vertical axis is the Z value of each variable. The box plot shows the Z value of each variable during model calculation. The green boxes represent important variables, and the red boxes represent unimportant variables


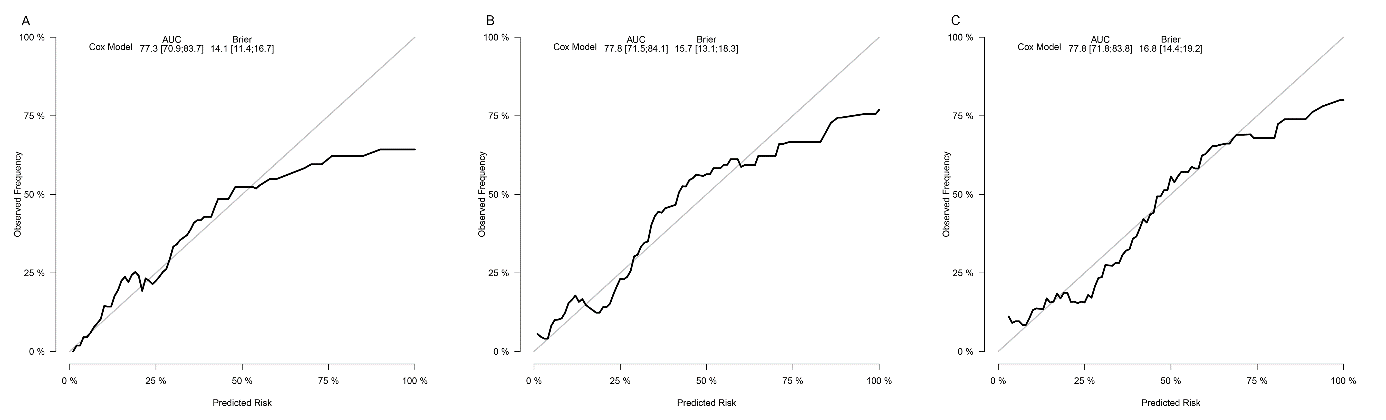


Figure S4. The calibration curves of prediction model for (A) 28 days, (B) 90 days, and (C) 365 days all-cause mortality; AUC: area under the curve; CI: confidence interval.


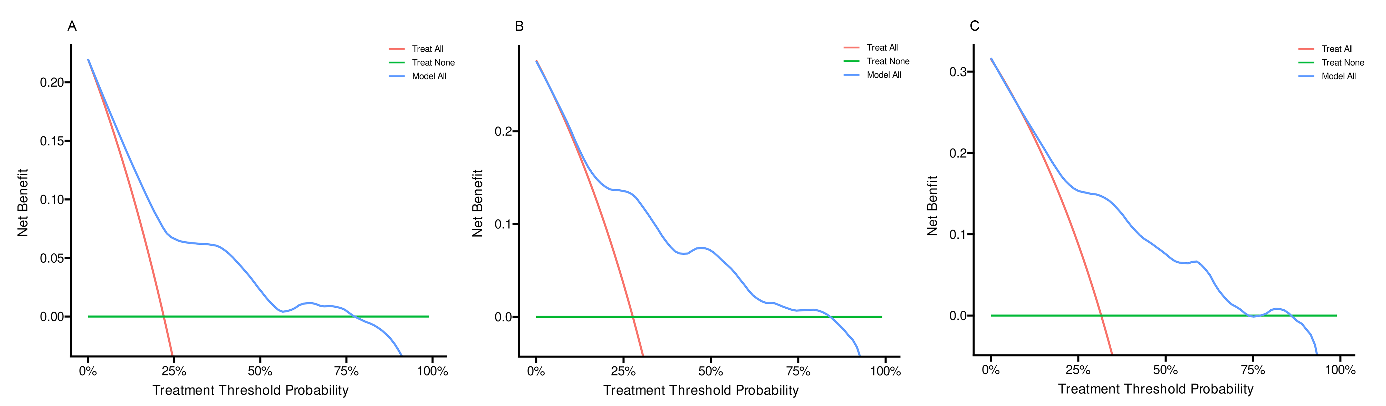


Figure S5. The DCA curves of prediction model for (A) 28 days, (B) 90 days, and (C) 365 days all-cause mortality; AUC: area under the curve; CI: confidence interval.
